# Antisepsis from the Later Standpoint

**Published:** 1884-04

**Authors:** 


### Editorial.

---

### ANTISEPSIS FROM THE LATER STANDPOINT.

It is within the memory of most medical men that for a healthy healing process a certain degree of inflammatory action was regarded as the main requisite in and about a wound. The only provision was that the inflammatory process should be limited in degree. Such expressions as " the accompanying inflammation will soon glue the parts together ;" and " all wounds must inflame," were often used as descriptive of the natural history of wound repair. But antiseptic surgery has shown that the slightest process of inflammation in wounds, is the cause of delay in repair. The redness, heat, œdema, and pain are only evidences of putrefaction and fermentation of the wound discharges. They are the protests of nature against the entrance of this material into the general circulation. With the fermentation come myriads of micro-organisms of the bacterial order, the latter related to the former as cause and effect. To-day, the surgeon's principal aim is to prevent this poisoning of a wound. He desires to secure healing without inflammation, without pus formation, without pain, and by first intention. This result is only attained by antiseptic practice. The method of antiseptic practice is now largely a matter of taste.

If the principle be faithfully and honestly kept in mind to prevent septic infection through the air surrounding the patient, the water with which his wounds are bathed, and the hands, instruments, and appliances by which the necessary surgical manipulations are made, the end is reached. It is, beyond question, the duty of every practitioner to render himself familiar with the technique of the processes and appliances necessary to render every wound aseptic. No better plan can be adopted than a careful study and familiarity with the details of Listerism, the purest and best of all antiseptic methods. From this method departures to those which are simpler can then be safely made. It is well enough to speak of cleanliness and perfect rest in wound treatment, but neither of these can be attained without absence of fermentation, absolute control of all hæmorrhage, perfect drainage, and close coaptation. The accumulation of wonderful results in surgical practice following the introduction and adoption of the principles of antiseptic surgery, as formulated by Mr. Joseph Lister, steadily increases. Compare the results of gunshot wounds of joints during our late war, and those obtained by Carl Reyher in the Russo-Turkish war. Of 18 patients reported by Reyher treated antiseptically, 15 recovered with mobile joints, 3 only dying. With us the rule was almost certain death or amputation. Reyher also gives 40 patients operated upon and manipulated without antiseptic precautions, with 34 deaths and 6 recoveries. Of these 5 lost their limbs. He also gives 23 patients left to themselves, all of whom died but one.

Nearly every text-book condemns the victims of penetrating gunshot wounds of joints to amputation of the limb. Antiseptic surgery and fixation, together with rigid abstention from disturbance of the wounds with septic fingers and probe save nearly all, and these frequently with mobile joints. It is here surely that the too inquisitive surgeon furnishes the straw which breaks the back of nature. These are only a few among a large number of revolutions in the management of dangerous wounds that have been wrought by this special practice, but these alone are so striking as to cause those to blush who still sneer at the notions of antiseptic work, and go on in the old method of slovenliness, dirt, and death. A Lister dressing is costly, and possibly cannot be adopted in universal practice. Many other methods safely take its place, and some are cheap, found everywhere, and capable of being used in the most adverse circumstances with such materials as aseptic sawdust, shavings, sugar, dried earth, and other absorbent substances. A solution of corrosive sublimate 1 to 2,000 ($7\frac{1}{2}$ grs. to water 1 qt.) thoroughly incorporated with any of these substances will furnish an absorbent antiseptic dressing sufficient to answer every purpose of cleaning wounds and prevention of fermentation and putrefaction changes with their long train of ills and woes. Equally good results often follow the use of the dry dressings with antiseptic cotton, or tow, or oakum. The only fact to be borne in mind is the necessity of recognizing the imperative importance of the aseptic condition and the adoption of the best methods circumstances will allow. When military field surgery, with antiseptic care and treatment presents a mortality of only 16 per cent. in the wounds heretofore most dangerous of all, the percentage of civil surgery ought certainly to be expressed by the single and smallest numeral.

---

A CERTAIN American was dyspeptic in London, and went to consult the great doctor, Abernethy, who was one of the brusquest men that ever lived, but a first-rate physician. "What's the matter with you?" asked Abernethy. "Why," replied the American, "I presume I have the dyspepsy." "Ah!" said he, "I see; a Yankee—swallowed more dollars and cents than he can digest." "I am an American citizen," was the reply, "and I am Secretary of Legation at the Court of St. James." "When did you arrive?" "Three days ago." "Ah! then you will soon be cured of your dyspepsia." "How so?" "Well, in the company you'll be obliged to keep you'll have to eat like a Christian." The gentleman did not know Abernethy's peculiarities, and made some sharp reply, on which Abernethy broke out: "I'll be hanged if I ever saw a Yankee that didn't bolt his food whole like a boa constrictor. How the d—l can you expect to digest food that you neither take the trouble to dissect nor the time to masticate? It's no wonder that you lose your teeth, for you never use them—nor your digestion, for you overload it—nor your saliva, for you expend it on the carpets, instead of on your food. It's disgusting. You call it dyspepsia; I call it guzzling. If you take half the time to eat you do to drawl out your words, and chew your food half as well as you chew your tobacco, you would be well in a month." And Abernethy was right.—*Vanity Fair.*

